# One-year follow-up evaluation of radiological and respiratory findings and functional capacity in COVID-19 survivors without comorbidities

**DOI:** 10.1097/MD.0000000000033960

**Published:** 2023-06-02

**Authors:** Hamza Ogun, Merve Gül, Yasemin Akkoyunlu, Esat Hayat, Nuran Gökbulut, Bilge Sümbül, Handan Başel Karaçöp, İsmail Yurtsever, Ayşegül Yabaci, Abdullah Kansu, Fatmanur Okyaltirik

**Affiliations:** a Department of Chest Diseases, Medical Faculty, Bezmialem Vakif University, Istanbul, Turkey; b Department of Infectious Diseases, Medical Faculty, Bezmialem Vakif University, Istanbul, Turkey; c Department of Medical Microbiology, Medical Faculty, Bezmialem Vakif University, Istanbul, Turkey; d Department of Radiology, Medical Faculty, Bezmialem Vakif University, Istanbul, Turkey; e Department of Biostatistic, Medical Faculty, Bezmialem Vakif University, Istanbul, Turkey; f Department of Chest Diseases, Medical Faculty, Medipol University, Istanbul, Turkey.

**Keywords:** 6-minute walk test, coronavirus disease 2019, lung involvement percentage, radiological abnormality, spirometry

## Abstract

The aim of this study was to assess clinical findings, radiological data, pulmonary functions and physical capacity change over time and to investigate factors associated with radiological abnormalities after coronavirus disease 2019 (COVID-19) in non-comorbid patients. This prospective cohort study was conducted between April 2020 and June 2020. A total of 62 symptomatic in non-comorbid patients with COVID-19 pneumonia were included in the study. At baseline and the 2nd, 5th and 12th months, patients were scheduled for follow-up. Males represented 51.6% of the participants and overall mean age was 51.60 ± 12.45 years. The percentage of patients with radiological abnormalities at 2 months was significantly higher than at 5 months (*P* < .001). At 12 months, dyspnea frequency (*P* = .008), 6-minute walk test (6MWT) distance (*P* = .045), BORG-dyspnea (*P* < .001) and BORG-fatigue (*P* < .001) scores was significantly lower, while median SpO_2_ after 6MWT (*P* < .001) was significantly higher compared to results at 2 months. The presence of radiological abnormalities at 2 months was associated with the following values measured at 5 months: advanced age (*P* = .006), lung involvement at baseline (*P* = .046), low forced expiratory volume in 1 second (*P* = .018) and low forced vital capacity (*P* = .006). Even in COVID-19 patients without comorbidities, control computed tomography at 2 months and pulmonary rehabilitation may be beneficial, especially in COVID-19 patients with advanced age and greater baseline lung involvement.

## 1. Introduction

The coronavirus disease 2019 (COVID-19) is still ongoing with various variants leading to peaks in patient counts.^[1,2]^ Globally, as of 23 September 2022, 611,421,786 confirmed cases of COVID-19 and 6,512,438 deaths have been reported by the World Health Organization.^[3]^ Although COVID-19 presents with a wide variety of clinical findings,^[4]^ pulmonary injury remains as the most common cause of morbidity and mortality associated with the disease.^[5,6]^ Greater severity increases the risk of hospitalization in the intensive care unit (ICU) and also causes long-term sequelae.^[7]^ Pulmonary sequelae may permanently affect the physical capacity and functions of individuals later in life; therefore, identifying risk factors that lead to lung injury and taking precautions for these risk factors can contribute to the management of COVID-19-related morbidity and mortality.^[2,8,9]^ Studies dealing with the longitudinal aspect of COVID-19 have mostly investigated dyspnea, radiological findings, pulmonary dysfunction and physical capacity impairment.^[5,10,11]^ Although not enough time has passed to enable conclusions regarding long-term effects, the adverse impacts on the lungs seem to last for months, years, and may perhaps become permanent.^[5,12]^

It is well known that the prognosis of COVID-19 is worse in patients with chronic comorbidities, particularly cardiovascular diseases, diabetes, chronic lung diseases and smoking.^[9,13–15]^ However, in most of the studies on the subject, patients with and without comorbidities have been evaluated together.^[12,16–18]^ To our knowledge, there are no studies investigating the radiological follow-up of lung injury or the changes in functional and pulmonary capacity in only individuals with COVID-19 who did not have comorbidities (non-comorbid patients, NCPs). Furthermore, in other studies including a variety of patients, follow-up periods were often short.^[2,5,8]^

In this study, we aimed to assess the frequency of dyspnea, radiological findings, pulmonary functions and physical capacity change over time among NCPs, and to investigate factors associated with the development of post-COVID-19 radiological abnormalities.

## 2. Methods

### 2.1. Study design and ethics

This prospective observational study was carried out between April 2020 and June 2020 at the Department of Chest Diseases, Bezmialem Vakif University Hospital, Istanbul, Turkey, according to the ethical standards stated in the Declaration of Helsinki and its later amendments. Ethical approval was obtained from the Ethics Committee of Bezmialem Vakif University Faculty of Medicine (date: May 5, 2020 and no: 54022451-050.05.04). Written informed consent forms were obtained from all the patients participating in the study.

### 2.2. Study population

**Figure 1.**
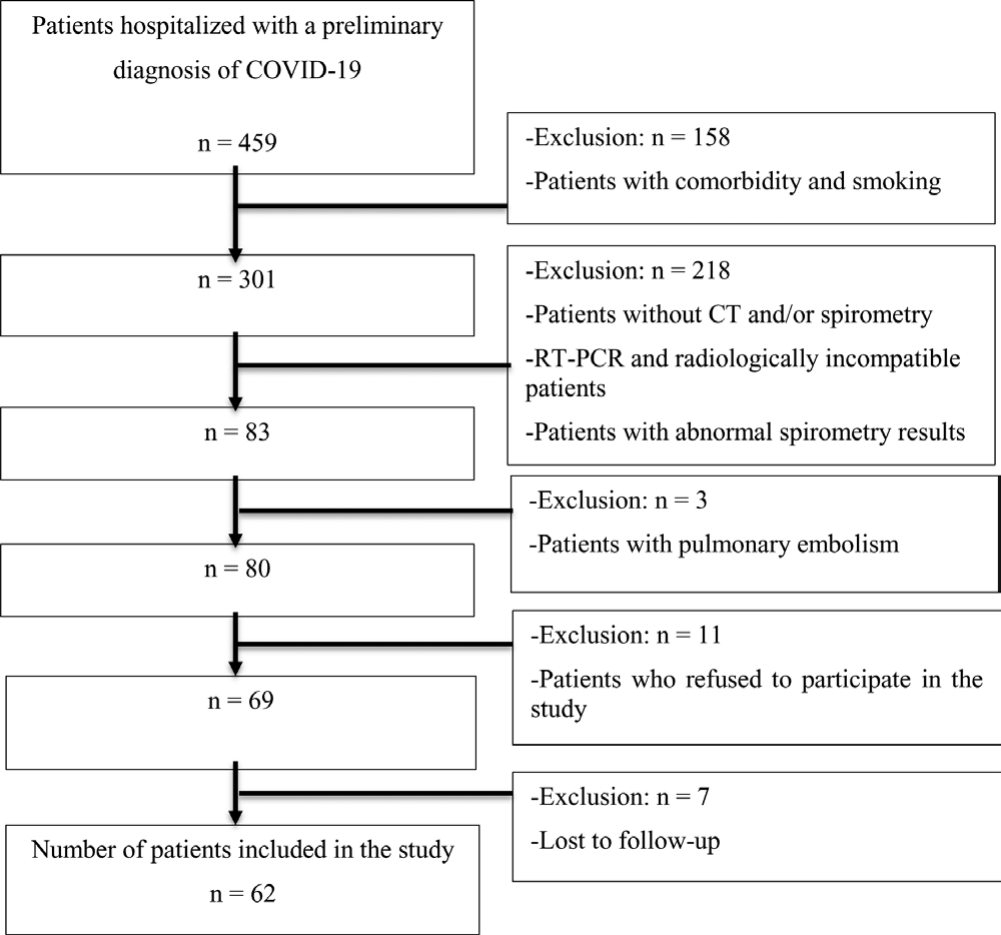
The flowchart of study. COVID-RADS = coronavirus disease 2019 imaging reporting and data system, CT = computed tomography, RT-PCR = real-time reverse transcription polymerase chain reaction.

A total of 62 NCPs with symptomatic disease who experienced moderate to critical COVID-19 pneumonia^[19]^ necessitating hospitalization were included in the study. COVID-19 was confirmed via real-time reverse transcription polymerase chain reaction (RT-PCR) positivity. The exclusion criteria were determined as follows: being < 18 or > 80 years old, not undergoing thorax computed tomography (CT) or spirometry at admission, having COVID-19 imaging reporting and data system (COVID-RADS) findings showing stage 2a or lower findings,^[20]^ having any known comorbidity (including prior abnormality in spirometry, mental illness, and any other infections/diseases that could alter radiological or functional results), having mild COVID-19 disease, smoking, and having suffered pulmonary embolism during COVID-19. We also did not include subjects who refused to participate in the study, those lost to follow-up, and patients who died during planned follow-up. The flowchart of the study is shown in Figure 1.

### 2.3. COVID-19 pneumonia management

The hospitalization, treatment, discharge and follow up management of COVID-19 pneumonia was carried out in accordance with the Turkish COVID-19 guidelines.^[21,22]^ After discharge, patients were scheduled for routine follow-up appointments (2nd, 5th and 12th months) in the outpatient clinic of the Department of Chest Diseases for at least 12 months.

### 2.4. Data collection and tools

At baseline, demographic characteristics such as age, sex, peripheral oxygen saturation (%) values (measured in ambient air), RT-PCR results, CT findings, blood test results, and psychiatric findings of the patients who met the inclusion criteria were recorded.

#### 1.2.4. Laboratory measurements.

At admission, swab samples were taken from the nasopharyngeal regions of the patients. A commercial test kit (Bio-Speedy SARS-COV2-2019-nCoV-qPCR Detection Kit; Bioeksen R&D Technologies, Istanbul, Turkey) was used for RT-PCR and all tests were performed according to the manufacturer’s instructions. Blood samples were acquired from the antecubital vein for the measurement of the complete blood count, D-dimer, ferritin and C-reactive protein. All laboratory measurements were performed via use of routine devices at the Clinical Microbiology and Biochemistry Laboratories of Bezmialem Vakif University Hospital.

#### 2.2.4. Pulmonary function test.

At baseline and at the follow-up assessment 5 months later (n = 55), spirometry was performed according to the European Respiratory Society (ERS)/American Thoracic Society (ATS) Task Guidelines^[23]^ using a portable spirometry device (Spirobank MIR®, Portable Spirometer A23, Rome, Italy) by trained spirometry technicians. The device was calibrated daily and sterilization was performed after each patient. The application was explained to the patients before the procedure. Obstruction was defined as FEV1/FVC (%) being below 70% and restriction was defined as FVC (%) being below 80% of references.^[24]^

#### 3.2.4. Radiological measurements.

All radiological evaluations were performed by an experienced radiologist. At admission, a 64-slice thorax CT (Aquilion CX; Toshiba Medical Systems, Tokyo, Japan) was performed with standard protocols regardless of RT-PCR result in patients with strong suspicion for COVID-19 pneumonia. Using the first CT images, COVID-RADS stage was determined for supporting the initial diagnosis and for determining the radiological severity of the disease at baseline. In this classification, patients are divided into 5 stages according to the level of COVID-19 suspicion (grade 0 and grade 1 low suspicion, grade 2A and 2B moderate suspicion, grade 3 high suspicion).^[20]^ Multifocal ground glass opacities (GGO) and GGO with superimposed consolidation were classified as grade 2B and 3. At baseline, moreover, we also assessed the average percentage of overall lung involvement according to the volumes of affected parenchyma using CT images. We evaluated each of the 5 lung lobes and the percentage of involvement in each lobe was determined visually. Then, the overall lung involvement percentage was calculated by taking the average of affected parenchyma volumes in each of the lobes.^[6,25]^

At the follow-up 2 months later, thorax CT was repeated (n = 50) and the patients were evaluated for the presence of radiological abnormalities by radiologist experience. Radiological abnormality was defined as the presence of at least one of the following: nodular opacities, pleural effusion, ground-glass opacities, consolidation, air bronchogram, pleural thickening, reticular pattern and bronchiectasis.^[2]^

At the follow-up 5 months later, standard posteroanterior and lateral lung X-ray (FCR XU-D1; Fujifilm, Tokyo, Japan) studies were performed for each patient using standard protocols. Thorax CT was performed only in patients with suspicious findings such as loss of aeration or increased opacity on X-ray (n = 9).

#### 4.2.4. Measures of physical capacity.

At the follow-up 2nd month (n = 58) and 12th month follow-up studies, the six-minute walk test (6MWT) was performed as recommended.^[26]^ Patients were asked to walk as much as possible for 6 minutes on a 30-meter-long straight walking track in an indoor area without supplemental oxygen. At the end of this period, the total distance walked was measured and recorded together with SpO_2_ and heart rate measured by a finger oximeter device.^[26]^ Before starting and at the end of the 6MWT, modified BORG scale was used for determination of the severity of the exercise-induced dyspnea (BORG-D) and fatigue (BORG-F). The modified BORG scale is a subjective scale consisting of numbers from 0 to 10 and verbal expressions that are used to describe increasing symptom intensity.^[27]^ Patients were asked to rate both BORG-D and BORG-F. The values at the end of the 6MWT were recorded and included in the analyses.

#### 5.2.4. Psychological assessment.

Psychological examination of the patients was performed at baseline by an experienced psychiatrist and they were evaluated for the presence of any psychological problems. In this context, depressive symptoms were detected in only 3 patients and these patients were found to have been prescribed antidepressants.

### 2.5. Statistical analysis

All analyses were performed on IBM SPSS Statistics for Windows, Version 25.0 (IBM Corp., Armonk, NY). For the normality check, the Shapiro–Wilk test was used. Data are given as mean ± standard deviation or median (1st quartile–3rd quartile) for continuous variables according to normality of distribution and as frequency (percentage) for categorical variables. Repeated measurements of continuous variables were analyzed with the paired *t* test or Wilcoxon signed ranks test depending on normality of distribution. Repeated measurements of categorical variables were analyzed with the McNemar test. Between groups analysis of continuous variables were performed with the independent samples *t* test or the Mann–Whitney *U* test depending on normality of distribution. Between groups analysis of categorical variables were performed with the chi-square test or Fisher’s exact test. Two-tailed *P* values of less than .05 were considered statistically significant.

### 2.6. Ethics statement

The present study protocol was reviewed and approved by the Institutional Review Board of Bezmialem Vakif University (approval No. 54022451-050.05.04-). Informed consent was submitted by all subjects when they were enrolled.

## 3. Results

51.6% of the participants were male and the mean age of all patients was 51.60 ± 12.45 years. The percentage of patients with radiological abnormalities at 2 months was significantly higher than at 5 months (*P* < .001). New-onset obstructive pulmonary dysfunction was detected in 5 (8%) patients. In 6 (9.6%) patients, new-onset restrictive-type respiratory dysfunction was detected. Data for all variables are summarized in Table 1.

**Table 1:** Summary of variables.

Age	51.60 ± 12.45
Sex	
Male	32 (51.6%)
Female	30 (48.4%)
Oxygen saturation, baseline (%)	
<90	29 (46.8%)
≥90	33 (53.2%)
RT-PCR, baseline	
Positive	52 (83.9%)
Negative	10 (16.1%)
COVID-RADS, baseline	
Grade 3	62 (100.0%)
Grade 2B	0 (0.0%)
Lung involvement percentage, baseline	
0–10%	31 (50.0%)
11–25%	18 (29.0%)
26–50%	11 (17.7%)
51–75%	2 (3.2%)
76–100%	0 (0.0%)
Neutrophil to lymphocyte ratio, baseline	3.0 (2.1–4.2)
D-dimer, baseline (µg/mL)	268 (140–378)
Ferritin, baseline (µg/L)	242 (120–498)
C-reactive protein, baseline (mg/L)	30.5 (13–67)
Psychological problem, baseline	
Absent	59 (95.2%)
Present	3 (4.8%)
Radiological abnormality, 2nd month*,†	
Present	22 (44.0%)
Absent	28 (56.0%)
Radiological abnormality, 5th month*,‡	
Present	4 (6.5%)
Absent	58 (93.5%)
FEV1, 5th month§	3.05 (2.40–3.64)
FVC, 5th month§	3.55 ± 0.93
FEV1/FVC (%), 5th month§	88 (83–90)

Data are given as mean ± standard deviation or median (1st quartile–3rd quartile) for continuous variables according to normality of distribution and as frequency (percentage) for categorical variables.

COVID-RADS = coronavirus disease 2019 imaging reporting and data system, FEV1 = forced expiratory volume in one second, FVC = forced vital capacity, RT-PCR = real-time reverse transcription polymerase chain reaction.

* Presence of radiological abnormality at 2nd vs 5th month *P* < .001.

† There are 12 missing data points.

‡ Computed tomography was required in 9 of the patients, radiological abnormalities were found in 4 of them.

§ There are 7 missing data points.

**Figure 2.**
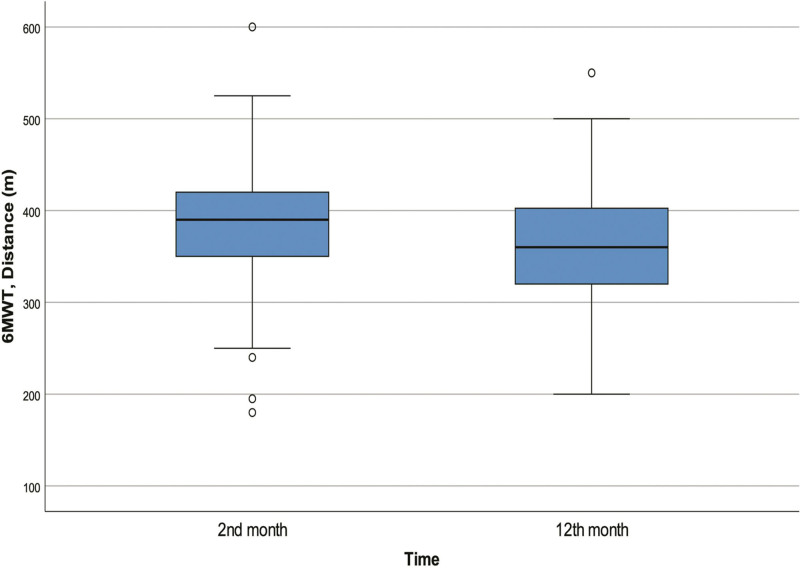
At the end of 2 and 12 months of 6MWT distance. 6MWT = 6-minute walk test.

The percentage of patients with dyspnea at 12 months was significantly reduced compared to 2 months (*P* = .008). The median SpO_2_ value after 6MWT was significantly higher at 12 months compared to that at 2 months (*P* < .001, Fig. 2); however, median walking distance was significantly shorter (*P* = .045). Median BORG-D (*P* < .001) and BORG-F (*P* < .001) scores after 6MWT at 12 months were significantly lower than at 2 months (Table 2).

**Table 2:** Summary of dyspnea, 6-minute walk test (6MWT) ad BORG scale scores.

		*P*
Presence of dyspnea		
2nd month	10 (16.1%)	**.008**
12th month	2 (3.2%)	
6MWT, SpO_2_ at final (%)		
2nd month*	96 (94–97)	**<.001**
12th month	97 (96–98)	
6MWT, Heart rate at final (bpm)		
2nd month*	96 (90–110)	.356
12th month	96 (90–102)	
6MWT, Distance (m)		
2nd month*	378.70 ± 82.90	**.045**
12th month	366.29 ± 72.97	
BORG-D score		
2nd month*	3 (2–4)	**<.001**
12th month	1 (1–2)	
BORG-F score		
2nd month*	3 (2–4)	**<.001**
12th month	1 (1–2)	

Data are given as mean ± standard deviation or median (1st quartile–3rd quartile) for continuous variables according to normality of distribution and as frequency (percentage) for categorical variables.

6MWT = 6-minute walk test, SpO_2_ = oxygen saturation.

* There are 4 missing data points.

**Figure 3.**
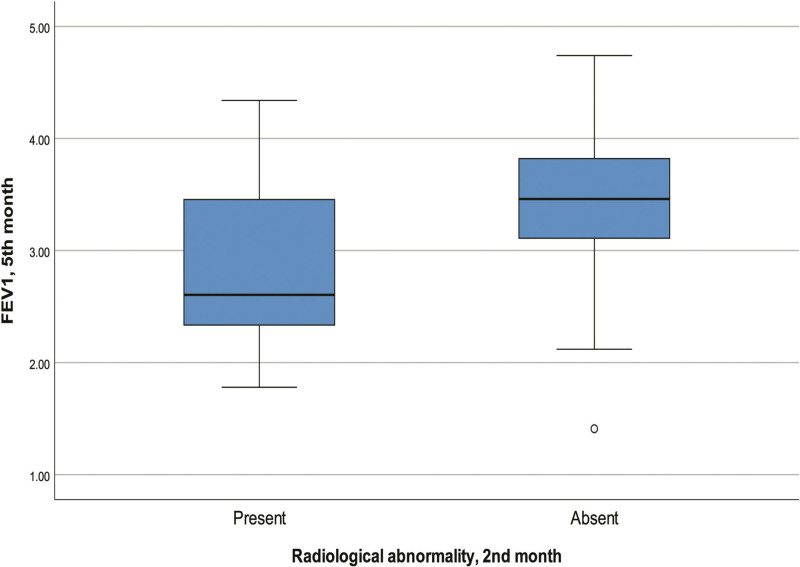
Radiological and FEV1 abnormalities at 2 and 5 months. FEV1 = forced expiratory volume in one second.

**Figure 4.**
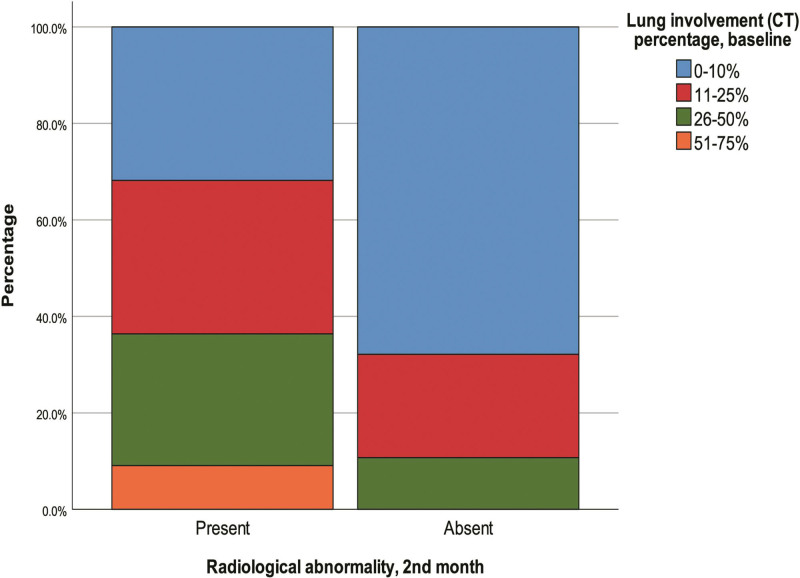
Presence/absence of radiological abnormalities at 2 months. CT = computed tomography.

According to univariate analyses, there was a significant correlation between the presence of radiological abnormalities at 2 months and advanced age (*P* = .006), low FEV1 (*P* = .018, Fig. 3) and low FVC (*P* = .006) levels at 5 months. Also, when patients were compared according to the presence/absence of radiological abnormalities at 2 months, we found that pulmonary involvement at baseline was significantly greater among those with radiological abnormalities at 2 months (*P* = .046, Fig. 4; Table 3).

**Table 3:** Summary of variables with regard to radiological abnormalities at the 2nd month.

	Radiological abnormality, 2nd month		*P*
	Present (n = 22)	Absent (n = 28)	
Age	56.95 ± 13.69	47.29 ± 9.85	**.006**
Sex			
Male	12 (54.5%)	15 (53.6%)	1.000
Female	10 (45.5%)	13 (46.4%)	
Oxygen saturation, baseline (%)			
<90	11 (50.0%)	13 (46.4%)	1.000
≥90	11 (50.0%)	15 (53.6%)	
RT-PCR, baseline			
Positive	19 (86.4%)	23 (82.1%)	1.000
Negative	3 (13.6%)	5 (17.9%)	
Lung involvement (CT) percentage, baseline			
0–10%	7 (31.8%)	19 (67.9%)	**.046**
11–25%	7 (31.8%)	6 (21.4%)	
26–50%	6 (27.3%)	3 (10.7%)	
51–75%	2 (9.1%)	0 (0.0%)	
76–100%	0 (0.0%)	0 (0.0%)	
Neutrophil to lymphocyte ratio, baseline	3.1 (2.3–3.9)	3.7 (2.05–4.95)	.457
D-dimer, baseline (µg/mL)	310.5 (163–548)	255 (140–336.5)	.177
Ferritin, baseline (µg/L)	302.5 (159–526)	167.5 (92.5–351)	.125
C-reactive protein, baseline (mg/L)	48 (23–82)	20 (12–69.5)	.190
Psychological problem, baseline			
Absent	21 (95.5%)	26 (92.9%)	1.000
Present	1 (4.5%)	2 (7.1%)	
FEV1, 5th month	2.61 (2.34–3.46)	3.46 (3.11–3.82)	**.018**
FVC, 5th month	3.27 ± 0.85	3.99 ± 0.82	**.006**
FEV1/FVC (%), 5th month	89 (83–90.5)	87 (83–90)	.492
Presence of dyspnea			
2nd month	2 (9.1%)	5 (17.9%)	.444
12th month	1 (4.5%)	0 (0.0%)	.440
6MWT, SpO_2_ at final (%)			
2nd month	95 (93–97)	96 (95–97)	.172
12th month	97 (96–98)	97 (97–98)	.204
6MWT, heart rate at final (bpm)			
2nd month	93.5 (90–98)	97 (90–110)	.328
12th month	94 (90–100)	96 (84.5–104.5)	.702
6MWT, distance (m)			
2nd month	361.25 ± 68.61	390.00 ± 87.56	.233
12th month	346.82 ± 56.01	386.07 ± 83.67	.053
BORG-D score			
2nd month	3 (2–4)	3 (2–4)	.783
12th month	1.5 (1–3)	1 (1–2)	.670
BORG-F score			
2nd month	3 (2–4)	3 (2–4)	.356
12th month	1.5 (1–3)	1 (0–2)	.147

Data are given as mean ± standard deviation or median (1st quartile–3rd quartile) for continuous variables according to normality of distribution and as frequency (percentage) for categorical variables

6MWT = 6-minute walk test, CT = computed tomography, FEV1 = forced expiratory volume in one second, FVC = forced vital capacity, RT-PCR = real-time reverse transcription polymerase chain reaction, SpO_2_ = oxygen saturation.

## 4. Discussion

The risk of persistent radiological abnormalities, pulmonary dysfunction and decreased physical capacity due to COVID-19 are especially higher in those with comorbid disease, thus most studies included patient groups with comorbidities.^[12,16,17]^ We investigated these risks in COVID-19 patients without comorbidities. As a result, we found that a significant proportion of the patients with radiological abnormalities in the 2nd month recovered by the 5th month. We observed that, at the 12th month, the number of patients with dyspnea, mean 6MWT distance, median BORG-D and BORG-F scores after 6MWT were significantly lower and median SpO_2_ at the end of 6MWT was significantly higher compared to the 2nd month. Moreover, a significant positive relationship was found between radiological abnormalities at 2 months and advanced age, and decreased FEV1 and FVC at 5 months.

Although most patients contracting COVID-19 survive, survivors are at risk of long-term sequelae involving multiple systems, particularly the respiratory system.^[12]^ Persistent inflammatory response is considered to be a key mediator in the formation of long-term sequelae.^[8,28]^ In this study, 44.0% of patients had radiological abnormalities at 2 months, which decreased to 6.5% at 5 months. In a prospective study, the baseline median CT severity score was significantly higher than that at 12 months, supporting radiological improvement.^[10]^ Another prospective study examined changes from baseline CT abnormalities after 2, 3, 6, and 12 months. The percentage of any abnormality at these time points were determined to be 76%, 63%, 67%, and 54%, respectively. The rate of recovery from chest CT abnormalities in the entire cohort was slower between the 1-year and 6-month follow-up studies compared to between 2 and 3 months.^[19]^ In the prospective cohort of Manolescu et al, according to the comparison of high-resolution CT (HRCT) imaging findings stratified by time intervals within 120 days after the first positive COVID-19 test, there was a significant increase in the percentage of patients with complete remission and a significant decrease in the percentage of patients with ground-glass opacities, trabeculation and other findings.^[2]^ Most post-COVID-19 radiological abnormalities resolve over time, but fibrotic changes can create permanent abnormalities.^[9]^ In this study, we found that the incidence of radiological abnormalities detected in the follow-ups of NCPs was lower than the incidences reported in other studies.^[2,19]^ This may be a predictable outcome, but it should be emphasized that even in NCP, the incidence of radiological abnormalities does not fall to zero. The lack of pre-COVID-19 radiological images of the patients may limit definitive interpretations, so more comprehensive studies are required for this patient group.

Dyspnea and associated loss of functional capacity are among the symptoms that worsen quality of life after COVID-19.^[29]^ The risk for dyspnea can be expected to be higher in those with chronic lung disease. In the present study, it was observed that dyspnea, post-exercise SpO_2_ level and fatigue complaints improved significantly among NCPs at 12 months compared to 2 months. But interestingly, the 6MWT distance decreased significantly at 12 months. In one study which collected data at admission and 6 weeks later, dyspnea was found to be present in 48% and 33% of patients, respectively.^[17]^ Other similar studies support that the percentage of patients reporting dyspnea demonstrate a decrease over time.^[11,30]^ Bellan et al compared data at baseline and at 4 and 12 months after disease. Although the percentage of patients complaining of dyspnea and fatigue decreased compared to baseline, they did not find a significant difference between the 4th and 12th months.^[10]^ Most studies have found that after SARS-CoV-2 infection, the 6MWT distance increases 3 to 6 months after the onset of infection.^[31,32]^ However, there are also studies reporting that the 6MWT values are similar at the 3rd and 24th months after COVID-19.^[33,34]^ It has been established that, compared to controls, patients with severe COVID-19 have worse 6MWT distance, SpO_2_ after 6MWT, BORG-F and BORG-D scores after discharge.^[35]^ According to the results of the present study, it can be said that there is a significant improvement in dyspnea and overall physical performance at the 12th month. However, although most NCPs recover completely, it should be kept in mind that there may be patients whose dyspnea complaints can continue for 12 months, and therefore, pulmonary rehabilitation might be needed even in this patient group.^[12]^

Investigating the factors associated with radiological abnormalities was another aim of this study. In this context, we compared patients with and without radiological abnormalities at the 2nd month follow-up. This analysis showed significant relationships with age and baseline lung involvement; however, other characteristics were similar. Additionally, since the number of patients with radiological abnormalities at 5 months was very small, statistical evaluations were not performed. In a retrospective study, it was shown that patients with fibrotic lesions in CT at follow-up (average 41.5 days) had significantly older age, greater CT severity score at baseline, higher ferritin, CRP, D-dimer levels, longer hospital stay, higher percentage of ICU requirement and higher percentage of steroid use than non-fibrotic patients.^[36]^ Multivariable analysis of a prospective study showed that age older than 60 years, initial critical COVID-19 severity and male sex were associated with persistent CT abnormalities at 1 year.^[19]^ In another prospective cohort with a large study group, the complete remission of lung CT abnormalities was found to be associated with a significantly higher average follow-up period and a significantly lower patient age.^[2]^ The positive results obtained in this study are in line with the literature. Higher lung CT severity scores in the acute phase was associated with worse disease severity.^[4,9]^ Therefore, it is highly likely that it is related to radiological abnormalities on follow-up. Already, the association of advanced age with post-COVID-19 radiological abnormalities has been shown in many studies.^[2,36,37]^ Elderly patients face a more serious COVID-19 disease and a higher risk of complications.^[38]^ However, this age-related finding of our study was unassociated with comorbidities, which is a notable finding of the present study. However, many other parameters investigated in this study were not associated with abnormalities on follow-up imaging. This may also be associated with the fact that the subjects of this study consisted of NCPs. More extensive studies are needed to confirm our findings.

Another important finding of the current study is that patients with radiological abnormalities at 2 months had significantly lower median FEV1 and FVC levels measured at 5 months. Klapholz et al^[8]^ reported a significant correlation between CT abnormalities and spirometry parameters measured 6 months later. In another study, patients with severe COVID-19 pneumonia had lower lung diffusing capacity for carbon monoxide/alveolar ventilation (DLCO)/VA), total lung capacity (TLC) and FVC at 4 months compared to those with mild pneumonia. Thus, they showed that the degree of lung injury during COVID-19 was associated with decreased respiratory function 4 months after acute infection.^[5]^ On the other hand, in another study, no significant correlation was found between lung total severity score and FEV1, FVC and FEV1/FVC values at 1 month after disease. However, of note, patients included in the latter study received pulmonary rehabilitation in the post-COVID-19 period.^[6]^ Impaired pulmonary function results may be due to severe inflammation in the acute phase and fibrous tissue in the later period.^[35]^ We show that not only the initial degree of lung damage, but the presence of lung damage 2 months after the acute phase can be associated with lung function in the later stages of recovery (month 5 in this study). Therefore, pulmonary rehabilitation programs may be considered in all patients, regardless of comorbidities or disease severity at baseline, in the post-COVID period. However, comprehensive studies with longer follow-up concerning pulmonary functions should be performed to determine the optimum time or duration of rehabilitation.

The most important feature that makes this study different from other similar studies is that the patient population consisted of only NCPs. In addition, it has a relatively longer follow-up period and evaluated radiological, clinical and functional results together. However, it has some limitations. It is a single-center study with a small number of patients. CT was requested from all patients in the 2nd month, but only from patients whose chest X-ray was suspicious at the 5th month. This may have caused an overestimation of patients with radiological recovery at 5 months. Since we did not have pre-COVID-19 radiological data of the majority of patients, we could not make a precise distinction between old and new lesions. In addition, no separate evaluation was made according to the severity of the disease, the need for mechanical ventilation or ICU, and the variety of radiological abnormalities. Finally, other tests such as DLCO^[17]^ and plethysmography,^[5]^ which would provide information about pulmonary fibrosis, were not performed.

To conclude, the frequency of radiological abnormalities decreased significantly from the 2nd to the 5th month among NCPs who had suffered from COVID-19 pneumonia. Compared to the 2nd month follow-up findings, we found that dyspnea, post-exercise fatigue, dyspnea severity, and 6MWT distance decreased, while SpO_2_ level after exercise increased at 12 months. Radiological abnormalities seen at 2 months were associated with advanced age and the percentage of lung involvement at baseline. In addition, patients with radiological abnormalities in the 2nd month had lower FEV1 and FVC levels in the 5th month. Even if there is no comorbid disease, it may be valuable to recommend control CT at 2 months after COVID-19 pneumonia and to administer pulmonary rehabilitation, especially in patients with advanced age and greater lung involvement at baseline. More comprehensive studies with longer-term outcomes of post-COVID-19 are needed for more appropriate management of recovering NCPs with COVID-19.

## Author contributions

**Conceptualization:** Hamza Ogun, Yasemin Akkoyunlu, Fatmanur Okyaltirik.

**Data curation:** Merve Gül, Esat Hayat, Nuran Gökbulut, Handan Başel Karaçöp.

**Formal analysis:** Hamza Ogun, Abdullah Kansu.

**Methodology:** Hamza Ogun, Bilge Sümbül, İsmail Yurtsever.

**Software:** Aysegul Yabaci.

**Writing – original draft:** Hamza Ogun.

**Writing – review & editing:** Hamza Ogun, Fatmanur Okyaltirik.
